# Case report: Uncommon manifestations of Rosai-Dorfman disease in the liver mimicking HCC

**DOI:** 10.3389/fonc.2024.1408353

**Published:** 2024-06-28

**Authors:** Huipeng Ren, Hao Zhang, Qinyun Wan, Yuhui Pang, Hongzhe Tian, Zhuanqin Ren, Yuan Cai

**Affiliations:** ^1^ Department of Medical Imaging, Baoji Central Hospital, Baoji, China; ^2^ Department of Pathology, Baoji Central Hospital, Baoji, China; ^3^ Department of Medical Ultrasonics, Baoji Central Hospital, Baoji, China

**Keywords:** Rosai-Dorfman disease, histiocytosis, non-Langerhans cell histiocytosis, extranodal lesion, hepatic lesions

## Abstract

Rosai-Dorfman-Destombes disease (RDD) is a rare non-Langerhans cell histiocytosis (LCH) disorder characterized by systemic extranodal lesions. Common cases include skin lesions, whereas liver lesions are rare. This study presents a case of a 66-year-old woman with a solitary extranodal liver lesion who underwent successful surgical treatment followed by glucocorticoid therapy. The patient did not experience any symptoms before surgery. The liver lesion was incidentally discovered during a routine ultrasound examination. Enhanced CT scan revealed the lesion with the characteristic of washout, similar to primary hepatic cancer (HCC). CT scans of the head, neck, chest, and abdominal pelvis revealed no lymph node or other organ lesions. After surgery, the liver lesion was diagnosed as RDD, and subsequent whole-body examinations did not reveal any skin lesions. The definitive diagnosis was solid liver RDD in adults. Although there were no typical cases of bilateral cervical lymph node lesions, ultrasound and CT examinations promptly detected liver lesions, leading to the correct diagnosis through surgical resection. The findings from this case indicate that RDD can occur in rare extrasegmental areas, and the imaging characteristics of liver lesions are not specific, indicating the importance of avoiding delayed diagnosis.

## Introduction

Rosai–Dorfman–Destombes disease (RDD) is a rare non-Langerhans cell histiocytosis (LCH) disorder (1). The incidence of RDD is approximately 1 in 200000 people, and it can occur at any age (1). RDD primarily affects bilateral cervical lymph nodes, but approximately 43% of RDD cases affect extranodal organs. The pathogenesis of RDD is complex, as it can occur independently or in association with viral infections, tumors, and autoimmune diseases and be acquired through familial inheritance (1). Clinically, RDD exhibits various manifestations lacking significant specificity. The histological characteristics of RDD include presence of lymphocytes, plasma cells, and large histiocytes exhibiting Emperipolesis. The characteristic histiocytes observed in RDD cases are S100+, CD68+, CD163+ and CD1a- and CD207- (2).

Currently, there is no standardized treatment regimen for RDD (1). Lymph node and skin lesions are typically self-limiting and only require observation (1). However, extranodal lesions require intervention, with surgical resection being a highly effective method for isolated focal extranodal diseases. Patients with multifocal unresectable extranodal lesions can be treated using various methods including glucocorticoids, sirolimus, radiotherapy, chemotherapy, and immunomodulatory therapy (2).

Liver involvement in RDD is extremely rare (1), which, combined with the lack of distinct imaging features, often leads to misdiagnosis and delays diagnosis (3). Consequently, histopathology and immunohistochemistry play indispensable roles in the diagnosis of RDD.

This study presents a case of an elderly woman who was diagnosed with RDD after the successful resection of a single liver lesion. Skin lesions, lymph node involvement, or other organ lesions were not detected after comprehensive whole-body examination and CT scan. Although this was an atypical case lacking bilateral cervical lymph node lesions, with non-specific imaging findings of liver lesions, CT scans clearly revealed the presence of liver lesions without involvement of lymph node and other organ. Consequently, the patient was diagnosed with solid liver RDD and received glucocorticoid treatment. The CT scan findings provided crucial information for clinicians to choose surgical treatment, and subsequent initiation of hormone therapy.

## Case report

A 66-year-old woman presented at the Hepatobiliary Surgery Outpatient Department following the discovery of a mass in the left lobe of the liver during a physical examination. Examination and review of her medical history showed that the patient did not experience abdominal pain, vomiting, bloody or black stools. In addition, she did not report any significant changes in weight before attending the health facility. Approximately 21 years ago, the patient underwent surgical resection for a benign nodule in the right breast. Moreover, she had been managing hypertension with oral nifedipine sustained-release tablets for the past 20 years. The patient resided in China her entire life and was not exposed to harmful chemicals, radioactive substances, or toxins. Furthermore, she has no history of hepatitis, tuberculosis, or malaria.

**Figure 1:**
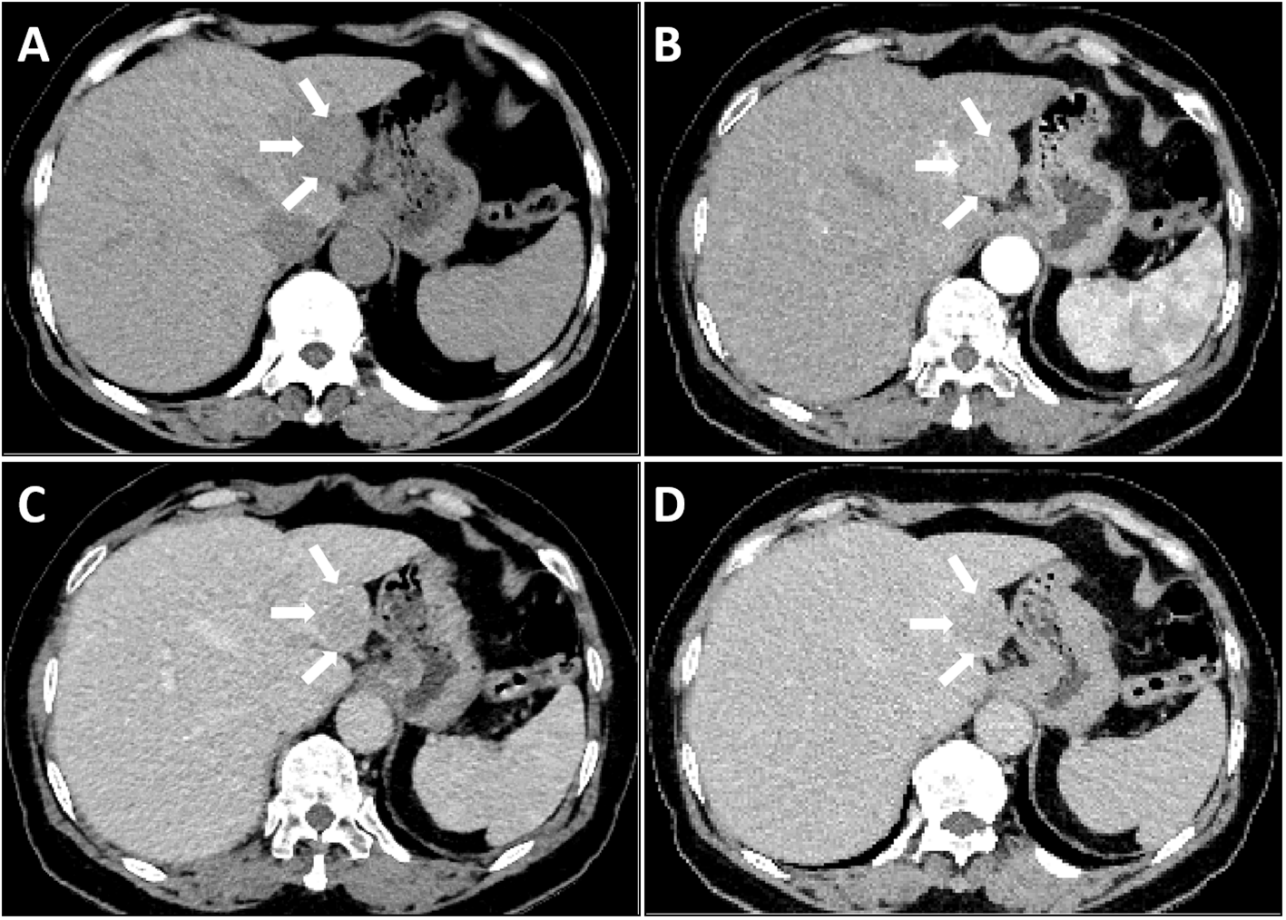
Liver CT scan: A single mass in the liver as observed by CT imaging (arrow). The Lesion exhibited slightly low-density on plain CT scan **(A)**, Mild enhancement was observed in the arterial phase **(B)**, Contrast washout observed in the portal phase **(C)** and delayed phase **(D)**.

Laboratory tests revealed a slight increase in total bilirubin and indirect bilirubin, In addition, the values of HBcAb and HCVAb are also relatively high. The levels of CEA, AFP, CA 12–5 and CA 19–9 markers were within normal limits, White blood cells are not high either. The above laboratory test results do not support the patient’s presence of hepatitis, tumors, and inflammation (**Table 1**). Subsequently, a CT scan of the upper abdomen revealed a spherical low-density mass measuring 3×3 cm in the left outer lobe of the liver, partially extending beyond the liver contour. The CT attenuation value of the mass ranged from 46–50 HU (**Figure 1A**). The CT attenuation value of the enhanced scan of the hepatic artery phase ranged from 75–86 HU (**Figure 1B**). In the hepatic portal vein phase scan, the CT attenuation value ranged from 72–81 HU (**Figure 1C**), whereas that of the delayed phase was approximately 66–78 HU (**Figure 1D**). This enhancement with contrast agent flushing enhances the effectiveness of detecting the possibility of primary HCC (4).

**Table 1:** Laboratory data on admission.

	Value	Normal range
Blood count		
White blood cells (×10^9^/L)	4.9	3.5–9.5
Neutrophil (%)	70.20	40–75
Lymphocyte (%)	21.50	20–50
Hemoglobin (g/L)	130	115–150
Platelet (×10^9^/L)	200	125–350
Urinalysis		
Protein	(−)	(−)
Occult blood	(−)	(−)
Serum chemistry		
total bilirubin (umol/L)	18.50	4.3–17.1
indirect bilirubin (umol/L)	13.70	1.7–10.2
Na(mmol/L)	140.0	137–147
K(mmol/L)	3.5	3.5–5.3
Cl(mmol/L)	102.7	99–110
Total protein (g/L)	79.0	65–85
Albumin (g/L)	45.8	40–55
Immunological findings		
HBsAg (IU/ml)	0.000	<0.05
HBsAb (IU/L)	263.080	<10
HBcAb(S/CO)	5.860	<1.0
HCVAb(S/CO)	0.070	<1.0
CA19–9(U/ml)	3.84	0–37
CA12–5(U/ml)	11.20	0–35
AFP(ng/ml)	2.38	0–8.78
CEA(ng/ml)	1.90	0–5

**Figure 2:**
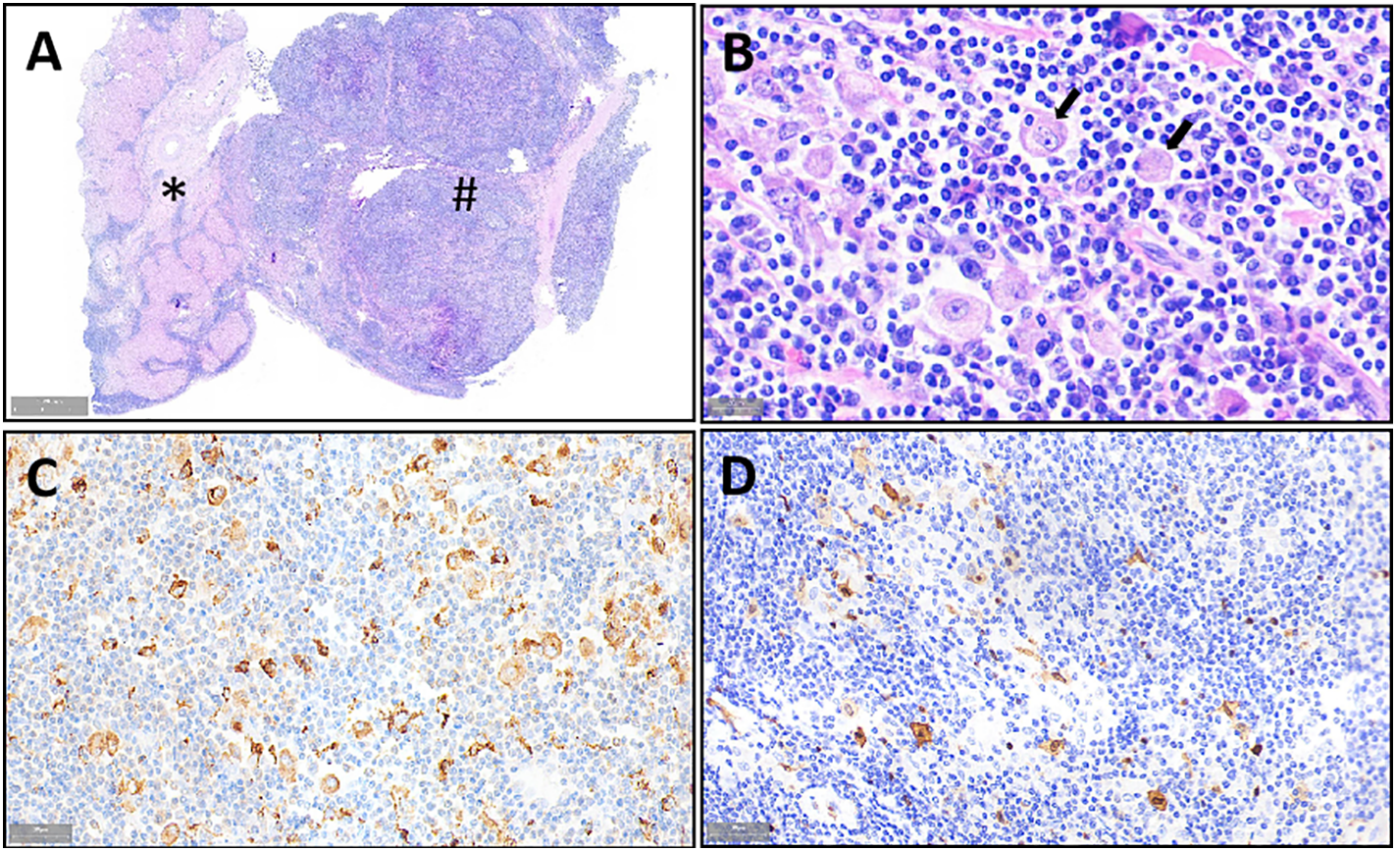
Morphologic and immunohistochemical features of RDD observed in the liver mass. Liver tissue (*) and diseased tissue (#) with high number of lymphocytes. **(A)** H&E staining image of large histiocytes distributed in the space between lymphoid follicles (×1 magnification), lymphocytes are observed in the cytoplasm of some large histiocytes (Emperipolesis) (indicated by the arrow). **(B)** H&E staining showing expression of CD68 **(C)** and S100 (×800 magnification), **(D)** Expression of CD68 **(C)** and S100 in the histiocytes as determined by IHC (×400 magnification).

The patient underwent minimally invasive laparoscopic surgery. Exploratory laparoscopy revealed the presence of a spherical tumor measuring approximately 3 cm in diameter, with a local protrusion on the left outer lobe of the liver. The tumor was located adjacent to the sagittal part of the portal vein, prompting the performance of a complete left hemihepatectomy to remove the tumor. Notably, liver cancer cells were not detected under a microscope. However, extensive proliferation of lymphoid tissue was observed, with lymph follicles of varying sizes evident within the tumor (**Figure 2A**). Furthermore, several large histiocytes with pale cytoplasm and oval nucleoli were observed in the interstitial zone of the follicles. Lymphocytes were observed in the cytoplasm of some large histiocytes, a phenomenon known as Emperipolesis (**Figure 2B**). Immunohistochemical analysis revealed positive expression of CD68, CD163, and S100, and negative expression of CD1a and OCT2 in the large histiocytes (**Figures 2C, D**). Morphological and immunohistochemical analyses confirmed the presence of histiocytosis except OCT2-, indicating a possible diagnosis of Rosai Dorfman Disease (RDD).

RDD is a rare disease that rarely occurs in the liver. According to Consensus recommendations for the diagnosis and clinical management of RDD (1), clinicians, pathologists, and radiologists had meticulous multidisciplinary discussions and decided to perform CT and ultrasonic (US) examinations of various organs and tissues, including the central nervous system, neck, chest, and pelvic cavity. In addition, a comprehensive examination of the entire body’s skin was conducted to classify and determine the extent of disease. The findings revealed the patient had no lymph node or other organ lesions, leading to a definitive diagnosis of solid liver RDD in adults.

The patient was treated with pharmacotherapy combined with systemic prednisone. Although no recurrent lesions were observed on CT scans three months post-diagnosis, a one-year follow-up was conducted, during which CT scans of the neck, chest, and abdomen were performed.

Despite lack of evidence supporting hepatitis and AFP positivity, the CT enhancement pattern characterized by “contrast washout” raised suspicion of HCC (4). The low incidence of the disease and the nonspecific nature of imaging findings pose challenges in accurately diagnosing hepatic RDD solely through preoperative imaging examinations.

Subsequently, the clinician prescribed glucocorticoid therapy and devised a follow-up plan for the patient.

## Discussion

This report presents a case of an elderly patient diagnosed with RDD and had a single lesion in the liver, exhibiting CT findings resembling the characteristics of hepatocellular carcinoma. The diagnosis was challenging due to the absence of lymph node, skin, or other organ lesions apart from the single liver lesion.

RDD is classified into familial, sporadic and skin types according to the classification of histiocytosis revised in 2016. Sporadic RDD is classified under group R, which is further divided into typical lymph node RDD, extranodal RDD, tumor-related RDD and immune disease-related RDD. Cutaneous RDD is categorized under group C, with sporadic RDD having the highest incidence (5).

RDD is predominantly reported in children and adolescents, but can occur at any age (1). However, a recent study reported that the median age at diagnosis for RDD was 50 years and another indicated that it was 56 years (6, 7). The patient in the present study was diagnosed with RDD at the age of 66 years.

Although approximately 43% cases involve extranodal organs, such as the skin, soft tissue, upper respiratory tract, bones and central nervous system, gastrointestinal involvement is rare occurring only in 1% of the cases (1). However, liver involvement is extremely rare (1). A previous study comprising 11 gastrointestinal patients showed that only 1 case exhibited liver involvement (8). Similarly, another study involving 11 gastrointestinal patients showed that 4 patients had liver lesions, with 1 patient having liver lesions exclusively, whereas the other 3 patients had lesions both inside and outside the liver (9). In the current case, the patient presented with a solitary liver mass without involvement of other organs.

In clinical settings, RDD exhibits various manifestations lacking distinct specificity. Nodal RDD typically manifests as painless enlarged lymph nodes in the neck regions, accompanied by symptoms such as fever, night sweats, and weight loss (2). Conversely, manifestation of extranodal RDD varies based on the affected organ. Skin RDD typically presents as slow-growing, painless, and non-itchy nodules or papules. Lesions in other organs typically result in mass effects, invading and damaging organ tissues, ultimately affecting organ function (1). Three case reports previously documented patients with liver RDD who presented with symptoms such as abdominal pain, fever or chronic nocturnal sweating (10–12). Furthermore, another case report highlighted a liver RDD patient who presented with generalized pain and progressive jaundice (13). However, in the present study, the patient did not exhibit symptoms such as abdominal pain or fever. The liver lesions were incidentally discovered during routine physical examinations, which is an extremely rare scenario.

Accurate diagnosis is essential for effective treatment of RDD. Although several imaging methods, including ultrasound (US), CT, magnetic resonance (MR), and positron emission tomography-computed tomography (PET-CT), are available for RDD diagnosis, the extremely low incidence of the disease and lack of distinct imaging features often cause confusion with other diseases, resulting in delayed diagnosis (3). Liver involvement in RDD is extremely rare, and there are limited reported imaging findings on liver lesions. The available reports of RDD cases indicate that liver lesions can manifest as solitary or multiple nodules, or as liver enlargement (3, 10–13). In previous studies, Contrast-enhanced CT imaging of liver RDD lesions revealed low enhancement (10–12). However, one case report indicated high enhancement of liver RDD lesions on Contrast-enhanced CT imaging (3). It is imperative to differentiate high enhancement lesions from hepatocellular carcinoma in a non-cirrhotic liver, whereas low enhancement lesions should be distinguished from intrahepatic cholangiocarcinoma, lymphoma, and metastasis. In the present case, an isolated hepatic mass, resembling HCC with “rapid contrast washout” on CT enhanced images, led the hepatobiliary physician to opt for surgical resection as the treatment approach.

Histopathology and immunohistochemistry play an indispensable role in RDD diagnosis due to lack of specific clinical features and imaging findings. The histological characteristics of lymph node RDD include alternating distribution of dark and bright areas between lymphocytes, plasma cells, and large pale histiocytes within the lesion. The nuclei of the large histiocytes typically exhibit an oval shape, vesicular structure, and prominent nucleoli. Emperipolesis, which allows lymphocytes to enter the histiocytes, is a useful feature in diagnosing RDD, although it is not specific as it can also be observed in conditions such as lymphoma, Erdheim-Chester disease (ECD), and LCH (2). Extranodal lesions resemble intranodal RDD lesions, but they exhibit increased fibrosis and low number of histiocytes, which are characteristic features of RDD. Histiocytes in RDD typically express S100, CD68, CD163, and OCT2, but do not express CD1a and CD207, unlike LCH (2, 14). The histopathology and immunohistochemistry findings of the present case were consistent with the diagnosis of RDD except OCT2-, OCT2- in RDD is very rare (14).

Currently, there is no standardized treatment regimen for RDD (1). Including observation, surgery, radiation therapy, and various drug treatments (2). MAPK pathway-targeted therapy has been experimentally used in treating some systemic RDD cases, but its effectiveness and safety require long-term observation and evaluation through multicenter prospective research (15). Interdisciplinary research involving clinical medical history, physical examination, imaging studies, laboratory tests, and other information can facilitate the development of personalized treatment approaches (1). Although the patient in this report was diagnosed with RDD only after undergoing surgical resection, the treatment met the requirements for isolated extranodal RDD. The patient promptly received glucocorticoid therapy without any delay in diagnosis and treatment. At the same time, the entire diagnosis and treatment process was also recognized by the patient, who stated that there was no physical discomfort.

In summary, this report presents a rare case of a single liver RDD resembling HCC in an elderly woman. CT scans distinctively revealed liver lesions while ruling out involvement of lymph nodes and other organs, providing important basis for doctors to opt for surgical treatment. Subsequent pathological examination confirmed the accurate diagnosis of RDD. These findings highlight the rare occurrence of RDD in the liver, and emphasize the non-specific nature of CT findings, indicating the importance of avoiding delays in diagnosis.

## Data availability statement

The datasets presented in this study can be found in online repositories. The names of the repository/repositories and accession number(s) can be found in the article/supplementary material.

## Ethics statement

The studies involving humans were approved by Ethics Committee of Baoji Central Hospital. The studies were conducted in accordance with the local legislation and institutional requirements. The participants provided their written informed consent to participate in this study. Written informed consent was obtained from the individual(s) for the publication of any potentially identifiable images or data included in this article.

## Author contributions

HR: Data curation, Writing – original draft, Writing – review & editing. HZ: Supervision, Writing – review & editing. QW: Project administration, Writing – review & editing. YP: Writing – review & editing. HT: Writing – review & editing. ZR: Writing – review & editing. YC: Resources, Supervision, Writing – review & editing.
